# Voltage stability assessment of grid connected PV systems with FACTS devices

**DOI:** 10.1038/s41598-022-26751-5

**Published:** 2022-12-24

**Authors:** Melat K. Abdullah, Lokman H. Hassan, M. Moghavvemi

**Affiliations:** 1 Department of Electrical and Computer Engineering, University of Duhok, Duhok, Kurdistan Region Iraq; 2 University of Science and Culture, Tehran, Iran; 3 Centre for Research in Applied Electronics (CRAE), Faculty of Engineering, University of Malaya, 50603 Kuala lumpur, Malaysia

**Keywords:** Electrical and electronic engineering, Energy infrastructure

## Abstract

Three static techniques (i.e. Power flow, Continuation Power Flow (CPF) and the Q–V curve) are used to assess the voltage stability of the power grid with a Solar Photovoltaic Generator (SPVG) and FACTS devices under nominal and heavy loading conditions. A static model is proposed for the power system that includes conventional power generation units and SPVGs with FACTS devices. Two models of SPVG were used (i.e., PV-model and PQ-model) to elucidate the effect of the SPVGs on the stability of the voltage under various operating conditions. The best location for FACTS devices was obtained under nominal and heavy load conditions using static techniques. A comparison between series and shunt FACTS devices under nominal and heavy loading conditions was carried out using the three static techniques. The interaction between SPVGs and FACTS devices was detailed in this paper. The proposed approach was tested on the New England 39-bus standard test system, and the results confirmed the effectiveness of the proposed approach under various operating conditions.

## Introduction

Voltage stability is the capability of a power grid at a specified initial operating condition to maintain steady voltages at all buses of the network under a disturbance. Voltage instability results in very low voltages in important parts of the network, culminating in partial or total blackout known as voltage collapse^1,2^.

Renewable energy sources, such as Solar Photovoltaic Generators (SPVGs), play an essential role in providing clean energy and ensuring adquate supply to meet energy demands. SPVGs can also be used to inject reactive power to the grid. However, the presence of SPVG in a network can cause system/voltage instability^3,4^, which makes imperative that voltage stability be accounted for when connecting SPVG generators to the grid.

Dynamic and static are two approaches mentioned in the literature for investigating voltage stability of grids. The dynamic analysis techniques were used in^5,6^ to confirm that the photovoltaic system can boost the system’s power requirements. Hassan et al.^7^ conducted a full dynamic study of voltage stability impact on the IEEE 69-bus and IEEE 118-bus distribution grids with SPVGs. In^8^, the long-term voltage stability of the network was improved after combining SPVG and Nordic-32 bus network using the time-domain simulation. However, dynamic analysis methods are time-consuming and require burden computation.

Static techniques are regularly used to investigate voltage stability due to their simplicity and reasonable accuracy^9^. In^10^, the static characteristics of the SPVG emerging in a power grid was investigated using only the power flow technique. SPVG’s performance on voltage stability was modelled in^11^ and^12^ using 14-bus with three generators test system and the Ontario test system as case studies, respectively. Both investigations utilized only the Continuation Power Flow (CPF) method. The presence of SPVG improved system stability by increasing the loadability boundary. The CPF method used in^13^ analyzed the stability of the voltage of grid-connected SPVG power systems under heavy load condition. The Q–V curve method was used by Wang et al.^14^ to elucidate the impact of the SPVG on the static voltage stability of China’s Qinghai network, while^15^ determined the impact of the SPVG on voltage stability boundary in an islanded microgrid using the P–V curve method. It is clear from the literature that the impact of the SPVG on voltage stability was investigated using only one of the static techniques.

FACTS devices implementation into the grid provided a significant opportunity to modify alternating current (AC) transmission, boost or decrease the power flow in exact buses, and respond immediately to instability issues^16^. The introduction of FACTS device into the power grid improves the stability of the grid^17^.

Anbarasan and Sanavullah^18^ proposed the CPF method for examining the grid at normal and heavy loading conditions. A Static Synchronous Compensator (STATCOM) was installed at the weakest bus in the network, and it was pointed out that the voltage magnitude of the bus improved as long as the reactive power support is connected to the weakest bus. The V-Q sensitivity method was proposed by P. Prabhakar and A. Kumar^19^ to highlight the impacts of High-Voltage Direct Current (HVDC), Static Var Compensator (SVC), Thyristor-Controlled Series Capacitor (TCSC), and Static Synchronous Compensator (STATCOM) on voltage stability.

In^20^, an automatic distribution of FACTS devices and variable tuning were used to improve voltage stability using an adaptive evolutionary algorithm. D. A. Ingole and P. D. V. N. Gohokar^21^ modeled the Static Synchronous Series Compensator (SSSC) to improve the system’s voltage stability. A controllability index was proposed by B. K. Kumar et al.^22^ to determine the optimal location of three types of FACTS devices for damping the inter-area mode of oscillations. Dynamic methods were used in^21^ and^22^.

This paper details a complete study conducted to highlight the impacts of SPVG and FACTS devices in enhancing voltage stability of power systems using three static techniques (i.e. power flow, Q–V curve and CPF). An efficient algorithm was provided for voltage stability analysis considering several factors. These factors are load capacity, type and location of FACTS devices and control type of SPVG. Two models of SPVG were used (i.e. PV-model and PQ-model) to demonstrate the effect of SPVGs on the stability of the voltage under various operating conditions. The optimum location for FACTS devices was determined under nominal and heavy load conditions. Power flow with Q–V curve techniques is used for nominal load condition. Also, continuation power flow with Q–V curve techniques is used for heavy load condition. The obtained results from first approach have been verified by using second approach. A comparison between series and shunt FACTS devices under both loading conditions was also carried out. The intercommunication between SPVGs and FACTS devices is detailed in this paper. The results confirmed the effectiveness of the suggested approach when examined on the New England 39-bus standard test system under various operating conditions.

## Modelling of solar photovoltaic generations with FACTS devices

This section details the modelling of SPVGs with FACTS devices.

### Modelling of the SPVGs

**Figure 1:**
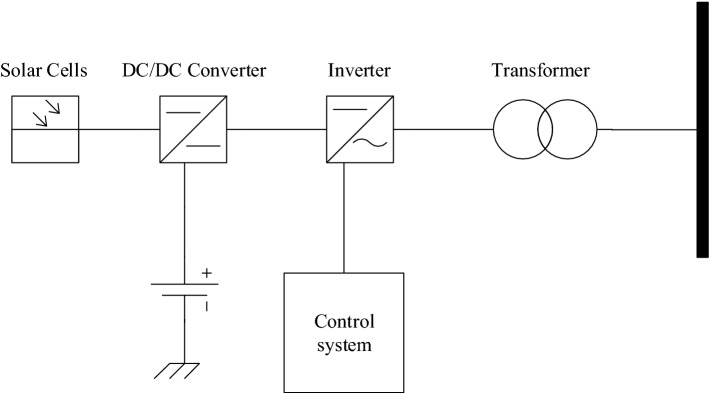
Structure of a grid-connected SPVG.

Figure 1 demonstrates the characteristic structure of a grid connected to a photovoltaic generator.

In this paper, two SPVG models are considered as:

- PQ-model when the active and reactive powers are constant control.
- PV-model when the active power and voltage are constant control.

The SPVG consists of photovoltaic arrays, a DC/DC converter, and an inverter. The outputs of the inverter are^5^:

1 $${i}_{d}=\frac{1}{1+s{T}_{p}}{i}_{d s},$$

2 $${i}_{q}=\frac{1}{1+s{T}_{q}}{i}_{q s},$$

where ${i}_{d}$, ${i}_{q}$ are the output currents of the inverter; ${T}_{p} , {T}_{q}$ are the steady-state gains and ${i}_{d s} ,$ ${i}_{q s}$ are the currents set-point.

The set-point currents can be calculated based on the chosen active power and reactive powers using the equation below:

3 $$\left[\begin{array}{c}{i}_{d s}\\ {i}_{q s }\end{array}\right]={\left[\begin{array}{cc}{v}_{d}& {v}_{q}\\ {v}_{q}& {-v}_{d}\end{array}\right]}^{-1}\left[\begin{array}{c}P\\ Q\end{array}\right],$$

where ${v}_{d}$ and ${v}_{q}$ are the output voltages of the inverter.

The PV model can be obtained by adding a voltage regulator to the PQ model. In the PV model, the value of the reactive power reference is calculated based on the value of the actual and set-point voltage via the PI controller using the equation below:

4 $${\text{Q}}= ({k}_{v}+{k}_{i}s)\left({v}_{dc}-{v}_{dc ref}\right),$$

where ${k}_{v}$, ${k}_{i}$ are the voltage PI controller gains.

### Modeling of FACTS devices

This work used classes of FACTS controllers (i.e. SVC, STATCOM and TCSC).

#### Static Var Componsator (SVC)

**Figure 2:**
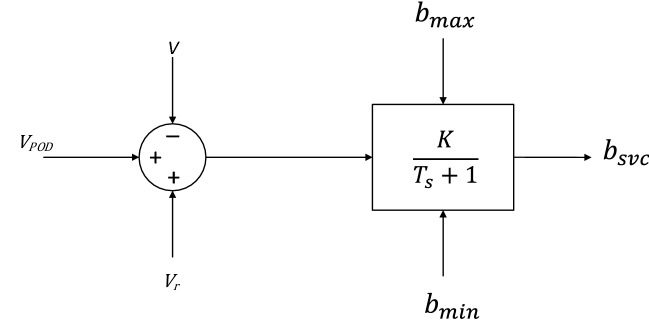
SVC model.

SVC as shunt FACTS device offers dynamically flexible shunt impedance to control the bus voltage. Figure 2 shows the model of SVC used in this work^23^.

The SVC role in a system is to recompense the required amount of reactive power by generating reactive power at the connected bus. The total susceptance ${\widehat{b}}_{svc}$ is given by^23^

5 $${\widehat{b}}_{svc}=(K{(V}_{r}+{v}_{POD}-V)-{b}_{svc})/T$$

Then, the reactive power generated by the SVC is:

6 $$Q={b}_{svc}{V}^{2},$$

where $K$ is the gain, ${V}_{r}$ is the voltage reference, ${v}_{POD}$ is the voltage of the power oscillation damping and $T$ is the time constant.

#### Static Synchronous Compensator (STATCOM)

**Figure 3:**
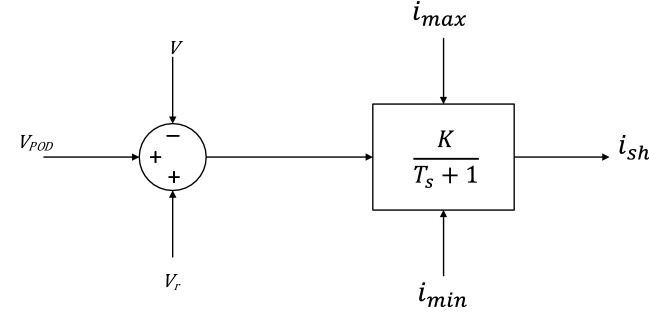
STATCOM model.

STATCOM is similar to SVC in providing shunt compensation but uses the voltage source converter. Therefore, it combines a very high content of power electronics. The implemented STATCOM model is a current booster model and the STATCOM current is kept in quadrature with the voltage of bus so that only reactive power is swapped between the STATCOM and AC system. Figure 3 shows the STATCOM model^23^.

The equations of the current and reactive power generated by the STATCOM are:

7 $${\widehat{i}}_{sh}=(K{(V}_{r}+{v}_{POD}-V)-{i}_{sh})/T$$

8 $$Q={i}_{sh}V$$

#### Thyristor-Controlled Series Capacitor (TCSC)

The model of a TCSC connected between bus $k$ and bus $m$ can be expressed as:

9 $${P}_{km}={V}_{k}{V}_{m}({Y}_{k}+B)\mathrm{sin}\left({\theta }_{k}-{\theta }_{m}\right)=- {P}_{mk},$$

10 $${Q}_{km}={V}_{k}^{2}\left({Y}_{k}+B\right)-{V}_{k}{V}_{m}({Y}_{k}+B)\mathrm{cos}\left({\theta }_{k}-{\theta }_{m}\right),$$

where $k$ and $m$ are the sending and receiving bus indices, and ${Y}_{k}$ is the line admittance.

**Figure 4:**
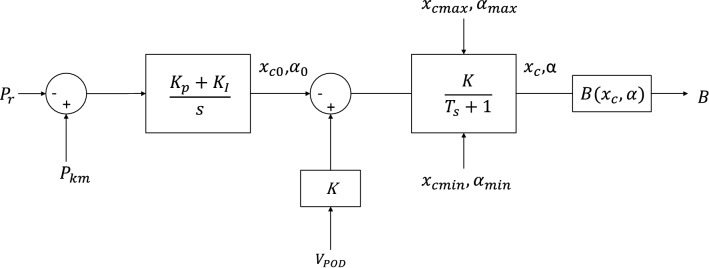
TCSC model.

The TCSC model is shown in Fig. 4^23^.

The state equation of the TCSC is:

11 $${\overrightarrow{x}}_{1}=(\left\{{x}_{c0},{\alpha }_{0}\right\}+K-{x}_{1})/T,$$

12 $${\overrightarrow{x}}_{2}={K}_{I}\left({P}_{km}-{P}_{r}\right),$$

13 $$\left\{{x}_{c0},{\alpha }_{0}\right\}={K}_{p}\left({P}_{km}-{P}_{r}\right)+{x}_{2},$$

where ${K}_{p}$ and ${K}_{I}$ are the proportional gain and the integral gain of the PI controller, respectively, ${x}_{c}$ is the reactance, $K$ is the gain of the stabilizing signal, and ${\alpha }_{0}$ is the firing angle.

The state variable ${x}_{1}=\left\{{x}_{c0},{\alpha }_{0}\right\}$ depends on the TCSC model and the PI controller is enabled during the constant power flow operation. The output signal is the series susceptance $B$ of the TCSC and can be defined as:

14 $$B\left({x}_{c}\right)= - \frac{{x}_{c}/{x}_{m}}{{x}_{km}(1-\frac{{x}_{c}}{{x}_{m}})}.$$

Through the power flow analysis, the TCSC is molded as a constant capacitive reactance, which adjusts the line reactance ${x}_{km}$ to:

15 $${\overrightarrow{x}}_{km}=(1-c){x}_{km}$$

where $c$ is the percentage of the series compensation.

## Voltage stability analysis

This section describes the three static analysis techniques for voltage stability assessment. These techniques (power flow, Q–V cure and CPF) were used to measure the stability of the voltage of the grid under various conditions.

### Power flow analysis

The electric utility industry depends on power flow as a static analysis to assess the voltage stability. The power flow analysis can be used to calculate the power flow via transmission line and voltage for buses or specific terminals. The system equations in term of the bus admittance matrix can be written as [1]:

16 $${\overrightarrow{I}}_{b}=\sum_{m=1}^{n}{\overrightarrow{Y}}_{bm} {\overrightarrow{V}}_{m},$$

where *Y* is admittance, $b$ is bus number, and $n$ is number of buses.

The current at any bus is related to active power, reactive power, and voltage via:

17 $${\overrightarrow{I}}_{b}=\frac{{P}_{b}-j{Q}_{b}}{{\overrightarrow{{V}^{*}}}_{b}},$$

where $P$ is active power and $Q$ is reactive power.

Then

18 $${P}_{b}+J{Q}_{b}={\overrightarrow{V}}_{b} \sum_{m=1}^{n}({G}_{bm}-J{B}_{bm}){\overrightarrow{{V}^{*}}}_{m},$$

where $G$ is conductance, $B$ is susceptance, and $Y=G\pm jB$.

Then, the active and reactive powers at each bus are the function of voltage magnitude and phase angle becomes:

19 $${P}_{b}={V}_{b}\sum_{m=1}^{n}({G}_{bm}\,{V}_{m}\,\mathrm{cos}{\theta }_{bm}+{B}_{bm}\,{V}_{m}\,\mathrm{sin}{\theta }_{bm}),$$

20 $${Q}_{b}={V}_{b}\sum_{m=1}^{n}({G}_{bm}\,{V}_{m}\,\mathrm{sin}{\theta }_{bm}-{B}_{bm}\,{V}_{m}\,\mathrm{cos}{\theta }_{bm}),$$

where ${\theta }_{bm}$ is the phase from bus to bus and equal to ${\theta }_{b}-{\theta }_{m}$.

### Q–V modal analysis

Voltage is fully associated with reactive power by assuming real power at every operating point is constant. The stability of the voltage can then be calculated via the incremental relation between reactive power and voltage^24^. The reduced Jacobian matrix becomes:

21 $$\Delta Q=J\Delta V.$$

Furthermore, Jacobian matrix can be factored as:

22 $$J=x\,{\Lambda }^{-1}\upeta ,$$

where $x$ is the right eigenvector matrix of Jacobian matrix, $\Lambda$ is the diagonal eigenvector matrix of Jacobian matrix, and $\upeta$ is the left eigenvector matrix of the Jacobian matrix.

The variation of voltage against the reactive power is:

23 $$\Delta V=x\,{\Lambda }^{-1}\,\upeta \,\Delta Q,$$

24 $$\Delta V=\sum_{i}{(x}_{i}{\upeta }_{i}/{\lambda }_{i})\Delta Q,$$

where ${x}_{i}$ is the ith column of the right eigenvalue of Jacobian matrix,${\upeta }_{i}$ is the ith row of the left eigenvalue of Jacobian matrix, and ${\lambda }_{i}$ is the ith eigenvalue of Jacobian matrix obtained from the diagonal matrix(${\Lambda }^{-1})$.

The voltage deviation for the *i*-th mode is:

25 $${V}_{i}=\frac{1}{{\lambda }_{i}} {q}_{i}$$

Eigenvalues of the Jacobian matrix can serve as an indicator of system’s voltage stability. The grid is stable when all the eigenvalues have positive values, or the grid is unstable when minimum eigenvalue is equal to zero or less than zero. Therefore, the lower value of positive eigenvalue, the nearer the system is to voltage instability. The V–Q sensitivity at $b$ bus is:

26 $$\frac{\partial {V}_{b}}{\partial {Q}_{b}}=\sum_{i}\frac{{x}_{bi} {\upeta }_{bi}}{{\lambda }_{i}}$$

A negative Q–V sensitivity means that the grid is unstable. This implies that lower sensitivity means a more stable grid. In order to determine the relation between the system buses and each eigenvalue, the Participation Factor (PF) becomes:

27 $${P}_{bi}={x}_{bi }{\upeta }_{bi}$$

### Continuation Power Flow (CPF) analysis

The continuation technique is a calculated path-following calculation procedure that can be used to calculate nonlinear equations for the system^25^. From Newton–Raphson method, load flow equations can be driven by introducing a load parameter into Eqs. (19) and (20).

Let $\gamma$ be the load factor, then:

28 $${P}_{Lb}={P}_{L0}+\gamma ({R}_{Lb}{S}_{base} \mathit{cos}{\theta }_{b}),$$

29 $${Q}_{Lb}={Q}_{L0}+\gamma ({R}_{Lb}{S}_{base} \mathit{sin}{\theta }_{b}),$$

where ${P}_{L0}$ and ${Q}_{L0}$ are nominal active and reactive load at bus $b$, ${R}_{Lb}$ is a multiplier to show the amount of load change at bus $b$ as load factor ($\gamma$) changes and ${S}_{base}$ is a given amount of apparent power, selected to provide suitable value of $\gamma .$

The equations of power flow becomes:

30 $$F\left(\theta ,\mathrm{v},\gamma \right)=0,$$

where $\theta$ represents the vector of bus voltage angles and $\mathrm{v}$ represents the vector of bus voltage magnitudes.

The active power generation term is adjusted as:

31 $${P}_{Gb}={P}_{G0}\left(1+\gamma {R}_{Gb}\right),$$

where ${P}_{Gb}$ is the active power generation at bus *b;* ${P}_{G0}$ is the initial value of active power generation; ${R}_{Gb}$ is the constant of varying rate in generation to resolve the problem.

A linear estimate technique was utilized by selecting a suitable step size in a direction tangent to the resolution path. Then, Eq. (30) becomes:

32 $${F}_{\theta }d\theta +{F}_{\mathrm{v}}d\mathrm{v}+{F}_{\upgamma }d\upgamma ={[F}_{\theta } {F}_{\mathrm{v}} {F}_{\upgamma }]\left[\begin{array}{c}d\theta \\ dv\\ d\gamma \end{array}\right]=0.$$

After adding $\upgamma$ to load flow equations, an additional equation is required to solve Eq. (32). This will be carried out by assuming one of the tangent vector components equal to + 1 or − 1, which is known as the parameter of the continuation.

33 $$\left[\begin{array}{c}{F}_{\theta }\\ \end{array}\begin{array}{c}{F}_{\mathrm{v}}\\ {e}_{b }\end{array}\begin{array}{c}{F}_{\gamma }\\ \end{array}\right]\left[\begin{array}{c}d\theta \\ dv\\ d\gamma \end{array}\right]=\left[\begin{array}{c}0\\ {}_{-}{}^{+}1\end{array}\right],$$

where ${e}_{b}$ is the suitable row vector with entirely elements equal to zero, excluding the ${b}^{th}$ element equals to 1.

First, $\upgamma$ is selected as the continuation parameter and as the process is continued the state variable with the highest amount of change is chosen as continuation parameter because of nature of parameterization. By solving Eq. (33), the tangent vector is obtained. The prediction is:

34 $${\left[\begin{array}{c}\theta \\ v\\ \gamma \end{array}\right]}^{p+1}={\left[\begin{array}{c}\theta \\ v\\ \gamma \end{array}\right]}^{p}+\sigma \left[\begin{array}{c}d\theta \\ dv\\ d\gamma \end{array}\right],$$

where the next step is designated by $p+1$ and the step size $\sigma$ is selected so the estimated solution is within the radius of the corrector convergence.

The predicted result is adjusted via local parameterization. Equation (33) is improved using one equation that identifies the amount of state variable, selected as:

35 $$\left[\begin{array}{c}F(\theta ,v,\gamma )\\ {x}_{b}-\eta \end{array}\right]=0,$$

where ${x}_{b}$ is state variable selected as continuation parameter and $\upeta$ is the predicted value of the state variable.

## The proposed approach

**Figure 5:**
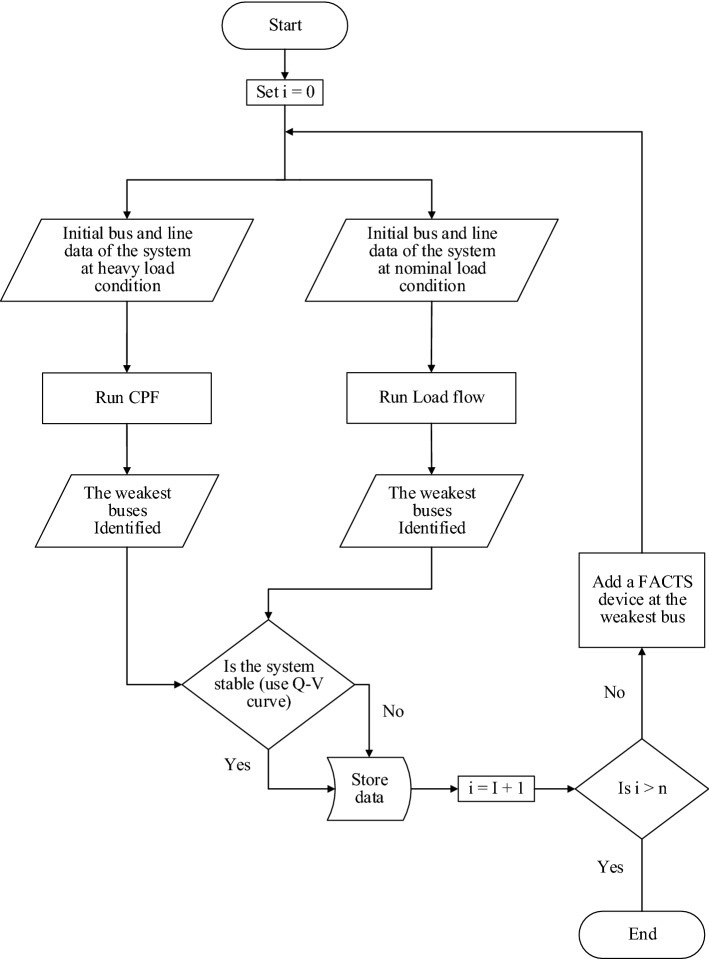
Flowchart of the proposed approach using three static techniques.

In this paper, three static techniques are applied to show the impact of SPVG or/ and FACTS devices on voltage stability of power grids. Also, the optimum location of FACTS devices in the power system with and without SPVG will be obtained under nominal and heavy load conditions. The proposed approach is illustrated in the flowchart in Fig. 5.

In Fig. 5, two cases are considered in the flowchart where the power system is considered to be with and without SPVG installed. As shown in the flowchart, after initialization, the algorithm first finds the weakest buses using load flow and CPF techniques at nominal and heavy load conditions, respectively. Then the stability of the system is tested under both operating conditions using Q–V curve technique. The algorithm then selects the weakest buses as the best location for the FACTS devices. After adding the FACTS device at the weakest bus, the process is repeated in order to obtain the current status of the system. If need arises the process can be repeated until the system monitoring parameters are within safe operating condition.

## Results and discussions

**Figure 6:**
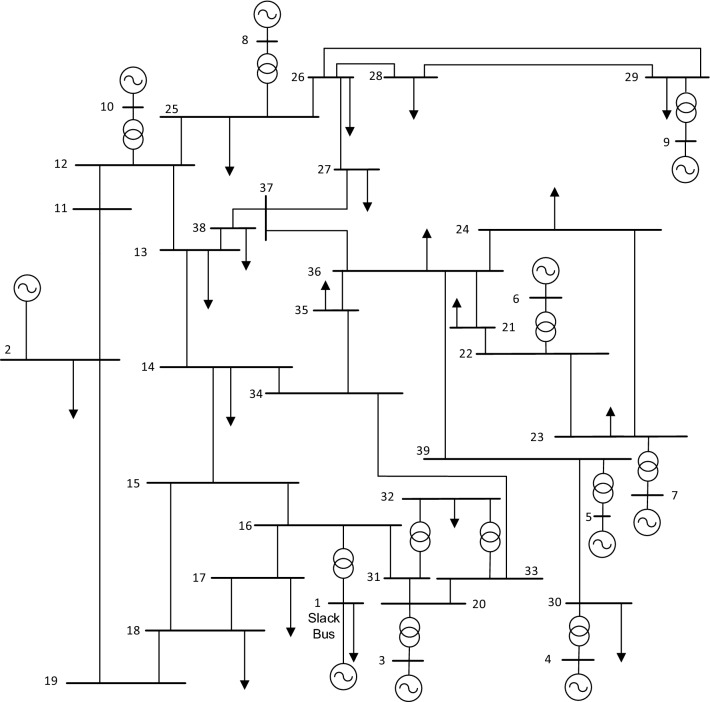
The single line diagram of the New England 39-bus system.

The New England 39-bus system was used to test the system. The single line diagram of the sysem is shown in Fig. 6. The study was carried out using Power System Analysis Toolbox (PSAT), which is a MATLAB-based toolbox for power system studies.

### The weakest bus

Power flow and the Q–V curve were used for nominal condition and CPF with the Q–V curve used for heavy load condition to identify the weakest buses of the system. The total power system losses was calculated for both conditions.

#### Nominal load condition

Table 1 shows three weakest buses based on the power flow results.

**Table 1:** Power flow results.

Bus	Nominal load		Heavy load	
	V [p.u.]	Phase rad	V [p.u.]	Phase rad
Bus 32	0.93491	− 0.11367	0.58693	− 0.50466
Bus 17	0.94407	− 0.18854	0.58128	− 0.84679
Bus 18	0.94479	− 0.19837	0.58496	− 0.90244

The weakest buses are bus 32 with voltage 0.93491 pu, followed by buses 17 and 18 with voltages 0.94407 pu and 0.94479 pu, respectively.

Using the Q–V curve technique, the eigenvalue of each bus was calculated and the three weakes buses listed in Table 2.

**Table 2:** Eigenvalue of weakest buses.

Associate bus	Nominal load		Heavy load	
	Eigenvalue	PF	Eigenvalue	PF
Bus 32	9.71090	0.10737		
Bus 27	19.1316	0.12076		
Bus 28	31.6577	0.31640		
Bus 17			− 0.77524	0.11414
Bus 24			8.24370	0.10055
Bus 32			19.3386	0.41880

The results confirmed that bus 32 is the weakest bus of the system because it has the lowest eigenvalue of 9.71.

#### Heavy load condition

Table 1 shows the three weakest buses per the CPF results.

Under heavy load condition, the weakest bus is bus17 with voltage 0.58128 pu followed by bus 18 with voltage 0.58496 pu and bus 32 with voltage 0.58693.

The eigenvalues of buses were calculated using Q–V curve technique and the results of only three weakest buses listed in Table 2.

By using the Q–V curve technique, the system becomes unstable at heavy load condition because one of the eigenvalues has a negative sign. The eigenvalue 16 linked to bus 17 is equal to − 0.77524.

### The optimum location of FACTS devices

The optimum location for the FACTS devices is at the weakest bus ^26^. In the previous section, buses 32 and 17 were identified as the weakest buses under nominal and heavy load conditions, respectively. This section details the optimum location for installing the SVC device (i.e. bus 32 or bus 17) using the proposed techniques. Here, buses 32 and 17 will be tested as the optimum location for the SVC.

#### SVC at bus 32

The power flow technique was applied under nominal load condition, and the calculated results are listed in Table 3.

**Table 3:** Power flow results when adding SVC at bus 32.

Bus	Nominal load p.u Phase rad		Heavy load	
	V [p.u.]	Phase rad	V [p.u.]	Phase rad
Bus 32	1	− 0.1146	0.62346	− 0.4893
Bus 17	0.95686	− 0.18508	0.60294	− 0.81322
Bus 18	0.95714	− 0.19462	0.60617	− 0.86555

**Figure 7:**
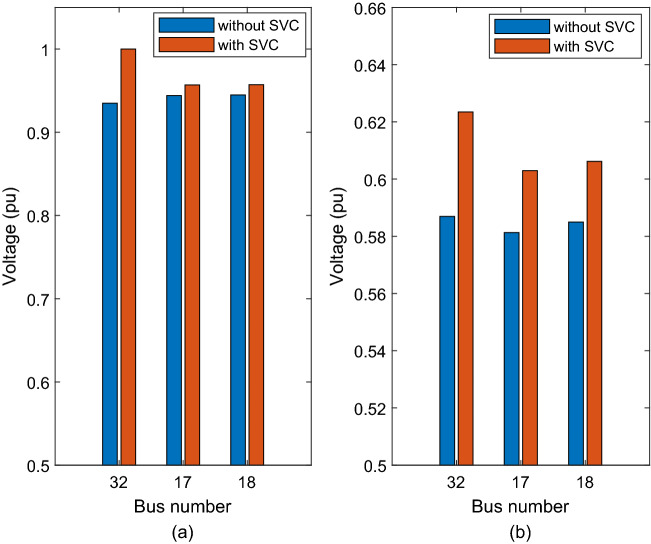
Voltage improvement after adding SVC at bus 32 (**a**) nominal load (**b**) heavy load.

After adding the SVC at bus 32, its voltage increased to 1 pu, bus 17 voltage increased to 0.95686 pu, and bus 18 voltage to 0.95714 pu as shown in Fig. 7a. The improvement was due to the SVC consisting of reactive power amounting to 2.025 pu at bus 32.

The CPF technique was applied under heavy load condition, and the results are listed in Table 3.

After installing the SVC at bus 32, the voltage of bus 17 increased to 0.60294 pu, voltage of bus 18 increased to 0.60617 pu, and the voltage of bus 32 increased to 0.62346 pu (see Fig. 7b).

The Q–V curve technique was used to check the stability of the system, and the results show that there is no eigenvalue with a negative sign, which means that the sytem remains stable at heavy load condition.

#### SVC at bus 17

The power flow technique was applied under nominal load condition, and the results are listed in Table 4.

**Table 4:** Power flow results after adding SVC at bus 17.

Bus	Nominal load p.u Phase rad		Heavy load	
	V [p.u.]	Phase rad	V [p.u.]	Phase rad
Bus 32	0.95925	− 0.10871	0.59329	− 0.4993
Bus 17	0.99277	− 0.18912	0.59477	− 0.83479
Bus 18	0.95714	− 0.19462	0.59718	− 0.88864

After the addition of SVC at bus 17, its voltage increased to 0.95925 pu, bus 17 voltage’s increased to 1 pu, and bus 18’s voltage increased to 0.99277 pu as, and the SVC’s composed reactive power amounted to 3.9047 pu at bus 17.

The CPF technique was applied under heavy load condition and the results listed in Table 4.

After adding the SVC at bus 17, the voltage of bus 17 increased to 0.59477 pu, bus 18’s voltage increased to 0.59718 pu, and bus 32’s voltage increased to 0.59329 pu.

The system becomes unstable at heavy load condition using the Q–V curve technique because one of the eigenvalues is negative. The eigenvalue 16 related to bus 17 is − 0.95852.

From the results, it can be surmised that bus 17 is the optimal location for SVC under nominal load condition for improving bus voltage. However, heavy load condition connecting SVC to bus 32 resulted in better values compared to bus 17, where the sytem becomes unstable.

### 39-bus system with SPVGs

This section details the PQ-model and PV-model of SPVG used to determine voltage stability. These models were tested at different load conditions and the results compared, then the SVC was used to improve the voltage stability of the system via both models of SPVG. The generator at bus 10 was replaced by a SPVG using both models as it is the smallest conventional generator capacity in the network .

#### SPVG PQ-model

The power flow technique was applied to the system at nominal condition, and the results listed in Table 5.

**Table 5:** Power flow results when SPVG PQ-model is connected at bus 10.

Bus	Nominal load p.u. Phase rad		Heavy load	
	V [p.u.]	Phase rad	V [p.u.]	Phase rad
Bus 32	0.93491	− 0.11367	0.60762	− 0.58859
Bus 17	0.94407	− 0.18854	0.59568	− 0.8884
Bus 18	0.94479	− 0.19837	0.59968	− 0.94345

It can be seen that bus 32 is the weakest bus, with a voltage of 0.93491 pu, bus 17, with a voltage of 0.94407, and bus 18, with a voltage of 0.94479. The results are similar to those obtained when the ordinary generator is connected to bus 10. It can be surmised that the results are unaffected when the ordinary generator is replaced with SPVG PQ-model.

The eigenvalue of the weakest buses were calculated using the Q–V curve technique and listed in Table 6.

**Table 6:** Eigenvalue of weakest buses with SPVG PQ-model.

Associate bus	Nominal load		Heavy load	
	Eigenvalue	PF	Eigenvalue	PF
Bus 32	8.5834	0.07806		
Bus 32	17.496	0.12288		
Bus 10	28.8855	0.35897		
Bus 17			− 1.3587	0.09504
Bus 10			7.03980	0.15506
Bus 10			16.6785	0.32574

After adding the SPVG PQ-model at bus 10, the eigenvalues increased. Table 5 shows the results of the continuation power flow when the system is heavily loaded.

At heavy load condition, the weakest bus is bus 17, with a voltage of 0.59568 pu, followed by bus18, with a voltage of 0.59968 pu, and bus 32, with a voltage of 0.60762. The results show that the voltage buses increased after adding SPVG PQ-model at bus 10.

The system become unstable at maximum load condition using the Q–V curve technique because one of the eigenvalues is negative. The eigenvalue 17 related to bus 17 is − 1.3587. Table 6 shows the eigenvalues of the weakest buses.

#### SPVG PQ-model with SVC

SVC was connected to bus 32 to enhance voltage stability, which is the optimum location, per Section “The optimum location of FACTS devices”.

The power flow was applied to the system installed with SPVG PQ-model and SVC, and the results listed in Table 7.

**Table 7:** Power flow results of the system installed with SPVG PQ-model and SVC.

Bus	Nominal load p.u Phase rad		Heavy load	
	V [p.u.]	Phase rad	V [p.u.]	Phase rad
Bus 32	1	− 0.1146	0.63962	− 0.57671
Bus 17	0.95686	− 0.18508	0.61181	− 0.86445
Bus 18	0.95714	− 0.19462	0.61544	− 0.91715

**Figure 8:**
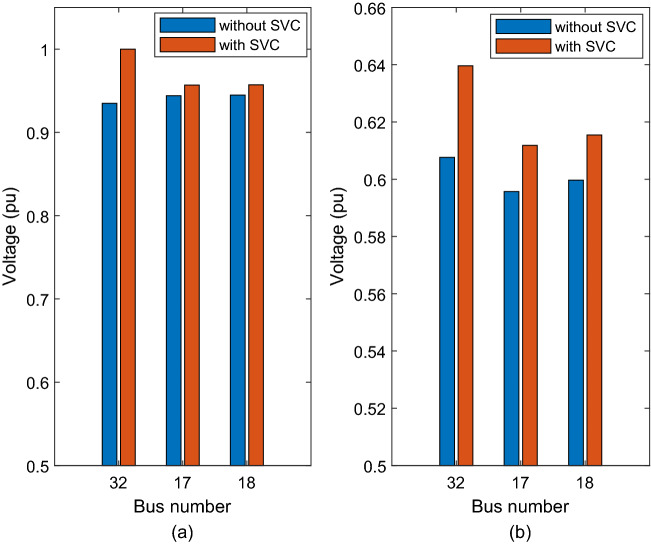
Voltage improvement after adding SVC at bus 32 (**a**) nominal load (**b**) heavy load.

The voltage at bus 32 increased to 1 pu, that of bus 17 increased to 0.95686 pu, and bus 18’s voltage increased to 0.95714 pu after the addition of SVC to the system as shown in Fig. 8. The voltage increased because the SVC’s composed reactive power amount of 2.025 pu at bus 32.

The continuation power flow was applied to the system under heavy load condition and the results listed in Table 7.

After adding the SVC at bus 32, bus 17’s voltage increased to 0.61181 pu, bus 18’s voltage increased to 0.61544 pu, and bus 32’s voltage increased to 0.63962 pu (see Fig. 8). The results showed clearly the impact of the SVC on improving the voltage profile at weakest buses (i.e. bus 17, bus 18 and bus 32).

The system remains unstable at maximum load even after adding SVC at bus 32 using the Q–V curve technique because one of the eigenvalues is negative. Eigenvalue 17 related to bus 17 is − 0.97003.

#### SPVG PV-model

At nominal load condition power flow was applied to the system, and the results of only three buses listed in Table 8.

**Table 8:** Power flow results of the system when SPVG PV-model is connected at bus 10.

Bus	Nominal load p.u Phase rad		Heavy load	
	V [p.u.]	Phase rad	V [p.u.]	Phase rad
Bus 32	0.93491	− 0.11367	0.62574	− 0.58461
Bus 17	0.94407	− 0.18854	0.61551	− 0.87518
Bus 18	0.94479	− 0.19837	0.61925	− 0.92807

**Figure 9:**
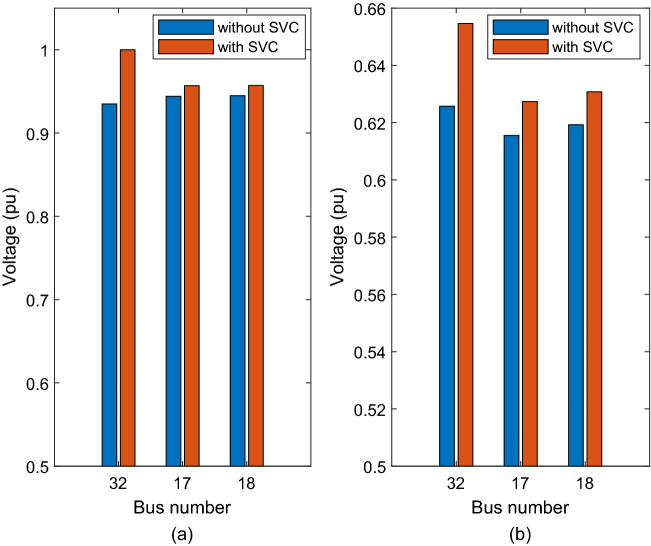
Voltage improvement after adding SVC at bus 32 (a) nominal load (b) heavy load.

The results show that the weakest bus is bus 32, with a voltage of 0.93491 pu, followed by bus 17, with a voltage of 0.94407 pu, and bus 18, with a voltage of 0.94479 pu (see Fig. 9).

The eigenvalue of each bus was calculated using the Q–V curve technique, and listed in Table 9.

**Table 9:** Eigenvalue of weakest buses of the system with SPVG PV-model.

Associate bus	Nominal load		Heavy load	
	Eigenvalue	PF	Eigenvalue	PF
Bus 32	8.5834	0.35897		
Bus 32	17.4960	0.07806		
Bus 10	28.8855	0.12288		
Bus 17			− 0.05095	0.1103
Bus 27			9.15790	0.10333
Bus 32			20.0631	0.10288

The eigenvalues increased after connecting the SPVG PV-model at bus 10. The weakest bus is bus 32, with an eigenvalue of 42. The buses voltage and total losses of the system at nominal load condition were unchanged after connecting the SPVG to the system.

The CPF was applied to the system when it is heavily loaded, and the results listed in Table 8.

The results confirmed that the weakest bus is bus 17, with a voltage of 0.61551 pu, followed by bus 18, with a voltage of 0.61925 pu, and bus 32, with a voltage of 0.62574. The voltages of the buses increased after using the SPVG PV-model.

After the SPVG PQ-model was used at bus 10, the buses voltage increased ~ 0.015 pu for each bus, but when SPVG PV-model was connected at bus 10, the buses voltage increased ~ 0.035 pu for each bus at heavy load condition. The results confirmed that the SPVG PV-model is more efficient than SPVG PQ-model in improving the system’s buses voltage under heavy load condition.

The system becomes unstable under heavy load condition using the Q–V curve technique because one of the eigenvalues is negative. The eigenvalue 18 related to bus 17 is − 0.05095. Table 9 shows the eigenvalues of the weakest buses.

It’s concluded that the system with the SPVG PV-model and with the SPVG PQ-model is unstable under heavy load condition.

#### SPVG PV-model with SVC

The power flow was applied to the system at nominal load condition and the results listed in Table 10.

**Table 10:** Power flow results of the system with SPVG PV-model and SVC.

Bus	Nominal load p.u Phase rad		Heavy load	
	V [p.u.]	Phase rad	V [p.u.]	Phase rad
Bus 32	1	− 0.1146	0.65464	− 0.57763
Bus 17	0.95686	− 0.18508	0.62733	− 0.85951
Bus 18	0.95714	− 0.19462	0.63077	− 0.91084

When the SVC was connected to the weakest bus, the voltage at bus 32 increased to 1 pu, bus 17 to 0.95686 pu, and bus 18 to 0.95714 pu because the SVC injects 2.025 pu reactive power to the system at bus 32.

The CPF was applied to the system at heavy loaded condition, and the results listed in Table 10.

The voltages of bus 17, bus 18 and bus 32 increased to 0.62733 pu, 0.63077 pu, and 0.65464, respectively, after the SVC was installed at bus 39 as shown in Fig. 9. The results showed the improvement of the voltage profile at the weakes buses.

The system is stable under heavy load condition applying the Q–V cure method, because all eigenvalues are positive.

### System with Shunt and Series FACTS Devices

Previous sections details the SVC as shunt FACTS device being used to improve the voltage stability of the system. This section describes the STATCOM as shunt FACTS device and TCSC as series FACTS device being used, and the results compared.

#### System with STATCOM

The power flow technique was applied to the system at nominal load codition, and the results listed in Table 11.

**Table 11:** Power flow result of the system with STATCOM installed.

Bus	Nominal load p.u Phase rad		Heavy load	
	V [p.u.]	Phase rad	V [p.u.]	Phase rad
Bus 32	1	− 0.1146	0.66152	− 0.59682
Bus 17	0.95686	− 0.18508	0.61649	− 0.88635
Bus 18	0.95714	− 0.19462	0.62005	− 0.93974

When STATCOM was used, the voltages at bus 32, bus 17, and bus 18 increased to 1 pu, 0.95686 pu, and 0.95714 pu, respectively.

From the results, it can be surmised that there are no differences between the results obtained using SVC and those obtained using STATCOM at nominal load condition.

The CPF was applied to the system under heavy load condition, and the results listed in Table 11.

When STATCOM was installed, the voltages of bus 17, bus 18, and bus 32 increased to 0.61649 pu, 0.62005 pu, and to 0.66152, respectively.

The system becomes unstable at heavy load condition using the Q–V curve technique because one of the eigenvalues is negative. The eigenvalue 17 related to bus 17 is − 0.15514.

The results show that SVC is more efficient that STATCOM in terms of bus voltage and stability.

### System with TCSC

The power flow technique was applied to the system when the load is nominal, and the results listed in Table 12.

**Table 12:** Power flow results of the system with TCSC installed.

Bus	Nominal load p.u Phase rad		Heavy load	
	V [p.u.]	Phase rad	V [p.u.]	Phase rad
Bus 32	0.94231	− 0.1141	0.55153	− 0.58443
Bus 17	0.94392	− 0.18857	0.62513	− 0.85446
Bus 18	0.94465	− 0.1984	0.62866	− 0.9058

After connecting the TCSC in series with line 31–32, the voltages at bus 32, bus 17, and bus 18 increased to 0.94231 pu, to 0.94392 pu, and 0.94465 pu, respectively.

**Figure 10:**
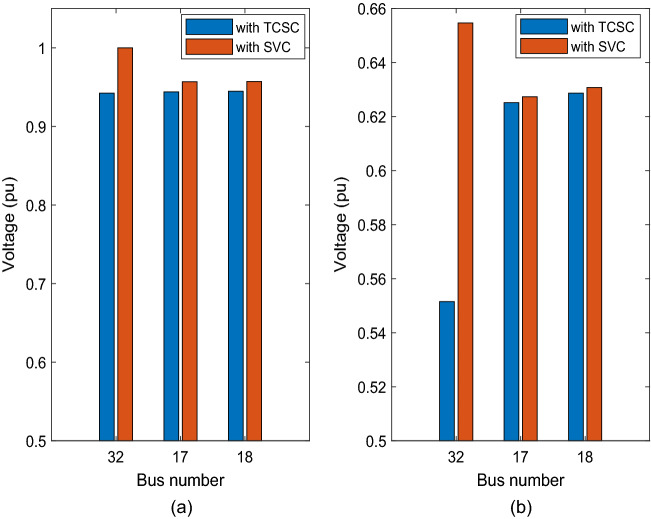
Voltage profile comparison when the system installed with TCSC and SVC (**a**) nominal load (**b**) heavy load.

The system with TCSC installed in line 31–32 at nominal load condition reported lower buses voltage when compared to SVC and STATCOM (see Fig. 10). Figure 10 shows a comparision between TCSC and SVC impact on improving the voltage profile at weakest buses.

The continuation power flow was applied at heavy load condition, and the results listed in Table 12.

The results show that the voltages of the bus 17, bus 18, and bus 32 increased to 0.62513 pu, 0.62866 pu, and 0.55153 pu, respectively, after TCSC was installed in line 31–32.

The system is stable under heavy load condition applying the Q–V curve when TCSC is connected in series with line 31–32.

The system with TCSC installed at heavy load condition reported lower buses voltage compared to the system with SVC or STATCOM installed (see Fig. 10). The system with TCSC installed is stable at heavy load condition, while the system with STATCOM installed is unstable.

## Conclusion

This paper detailed three static techniques used to assess the voltage stability of a power grid. The weakest bus in the grid under nominal and heavy loading conditions was obtained using the New England 39-bus system as a test system. These techniques are power flow, Q–V curve, and CPF. Bus 32 and bus 17 were identified as weakest buses under nominal load and heavy load conditions, respectively, and the system became unstable under heavy loading condition. FACTS devices were used to improve the voltage stability of the system. The optimum location of FACTS devices was obtained using three static techniques under both loading conditions. It was concluded that bus 32 is the optimal location for SVC device under heavy loading condition. The voltage of buses increased ~ 6.5 and ~ 3.6% at nominal load and heavy load conditions, respectively, when using FACTS devices.

PQ-model and PV-model for SPVG were used to assess the stability of voltage. These models were tested at different load conditions, and the results compared. Then, the FACTS devices were used to improve the voltage stability of the system with PQ-model and PV-model of SPVG. The generator at bus 10 was replaced by a SPVGs, and the voltage stability of system was enhanced after the addition of the SPVG to the system. The results showed that SPVG PV-model is more effective than SPVG PQ-model in improving the system’s voltage stability of the system, reporting an improvement of ~ 3%.

Finally, three types of FACTS devices (i.e. SVC, STATCOM and TCSC) were compared under nominal and heavy loading conditions using three static techniques. The results showed that the shunt FACTS devices are more effective than series one in improving the system voltage stability.
